# Stem Cell Treatment for Alzheimer’s Disease

**DOI:** 10.3390/ijms151019226

**Published:** 2014-10-23

**Authors:** Ming Li, Kequan Guo, Susumu Ikehara

**Affiliations:** 1 Department of Stem Cell Disorders, Kansai Medical University, 2-5-1 Shinmachi, Hirakata City, Osaka 573-1010, Japan; E-Mail: liming@hirakata.kmu.ac.jp; 2 Department of Cardiac Surgery, Beijing Institute of Heart, Lung & Blood Vessel Disease, Beijing Anzhen Hospital Affiliated to Capital Medical University, Beijing 100029, China; E-Mail: guokequan@hotmail.com

**Keywords:** Alzheimer’s disease, stem cell therapy, oxidative stress, neurodegeneration

## Abstract

Alzheimer’s disease (AD) is a progressive and neurodegenerative disorder that induces dementia in older people. It was first reported in 1907 by Alois Alzheimer, who characterized the disease as causing memory loss and cognitive impairment. Pathologic characteristics of AD are β-amyloid plaques, neurofibrillary tangles and neurodegeneration. Current therapies only target the relief of symptoms using various drugs, and do not cure the disease. Recently, stem cell therapy has been shown to be a potential approach to various diseases, including neurodegenerative disorders, and in this review, we focus on stem cell therapies for AD.

## 1. Introduction

Alzheimer’s disease (AD) is the most common form of dementia, which is one of the major causes of disability and dependency among older people worldwide. AD manifests as an impaired ability to comprehend or use words, poor coordination and gait, and impaired executive functions in the realms of planning, ordering and making judgments. Generally, classification of AD includes familial and sporadic AD. Familial AD presents mainly as the mutation of three genes: The amyloid precursor protein (APP), presenilin-1 (PS-1) and presenilin-2 [1]. Sporadic AD results from environmental factors and risk genes, with apolipoprotein (ApoE) reportedly the most important [2]. Pathologic characteristics of AD are β-amyloid (Aβ) plaques, neurofibrillary tangles (NFT) and neurodegeneration. Aβ peptide is the main constituent of senile plaques, and Aβ fibrils from pores in neurons have been shown to lead to calcium influx and neuronal death [3]. NFT consists of neurofibrillary protein aggregates, formed as abnormal hyperphosphorylation of tau protein, which is one of the microtubule-associated proteins [4]. These pathological changes lead to synaptic loss, neuronal dysfunction and death. Furthermore, microglia have been reported to play an important role in the immune defense system of the central nervous system (CNS). Microglia activation and the release of associated inflammatory factors has been reported to contribute to chronic neurodegenerative disorders in AD [5]. Decreasing amyloid deposits and the use of antioxidant therapies have some ability to alleviate AD and, more recently, cell therapy has been seen as a potential approach to its treatment. In this review, we focus on stem cell therapies for AD.

## 2. Pathophysiology of AD

The pathophysiology of AD includes loss of neurons and synapses in the cerebral cortex and parts of the subcortical areas [6]. APP is cleaved by α-, β- and γ-secretases, generating a soluble peptide in normal people, but APP generates Aβ through abnormal processing, such as in AD. Aβ oligomers are thought to be the most toxic due to their impairment of synaptic and neuronal functions, leading to neurodegeneration that is clinically manifested by memory and cognitive dysfunction [7]. Tau is a neuronal microtubule-associated protein, separated from microtubules when it is phosphorylated. It forms helical filaments and aggregates neurofibrillary tangles in the neuronal cytoplasm [8]. One report has indicated that Aβ may accelerate tau aggregation into NFT, and that tau reduction can block Aβ- and excitotoxin-induced neuronal dysfunction in the AD mouse model [9].

Chronic neuroinflammation is one of the major factors in the pathophysiology of AD [10], this disease being associated with an inflammatory response as a result of increasing numbers of activated microglia and astrocytes, and activated complement proteins, cytokines, and reactive oxygen [11]. Microglia may release inflammatory cytokines, chemokines, and growth factors to remedy neuronal injury, and while microglia remain in a resting state in the healthy adult brain, they undergo dramatic changes in morphology in response to injury [12,13]. Aged microglia show a higher level of activation, and release inflammatory cytokines such as IL-1, IL-6 and TNFα, which are associated with age-related cortical atrophy in humans [14]. Recent research suggests that astrocytes are not simply passive support cells for neurons, but are active participants in neural information processing in the brain. TGF-β was detected around and within senile plaques in brains from Alzheimer patients, indicating that Aβ deposits may also induce an anti-inflammatory effect. Moreover, astrocytes produce IL-6, resulting in a localized inflammatory state within the CNS, and anti-inflammatory drugs such as tenilsetam show beneficial effects in AD animal models, suggesting that anti-inflammation therapy may help prevent AD [10].

Oxidative stress is a sign of aging and an important pathogenic factor in AD. Oxygen metabolism generates free radicals such as hydroxylradical, superoxide radical, and reactive nitrogen species, inducing reactive oxygen species (ROS) [15]. An imbalance between oxidant and antioxidant agents could generate oxidative stress, which induces damage to macromolecules and disrupts the reduction/oxidation (redox) signaling [16]. Mitochondria contain many redox enzymes, and generate ROS as a result of inefficient oxidative phosphorylation. Mitochondrial dysfunction occurs early and has a primary role in the pathogenesis of AD [17]. Moreover, one report has shown that the transition metals such as Cu^2+^, Zn^2+^ and Fe^3+^ are associated with Aβ aggregation and oxidative damage in the AD brain, and that Aβ catalyses the reduction of Cu^2+^, Fe^3+^ and H_2_O_2_, leading to the formation of proapoptotic lipid peroxidation products [18]. Aβ, which accumulates in parenchyma and blood vessels causes microglial migration and promotes inflammatory responses. Aβ also decreases ATP and increases ROS generation in mitochondria, leading to cell apoptosis [19,20].

## 3. General Treatment for AD

Deposits of Aβ are a pathological hallmark of AD, and thus depleting Aβ should be a useful therapy for AD. Cathepsin B is a cysteine protease of the papain superfamily, and degrades peptides and proteins that enter the endolysosomal system by endocytosis or phagocytosis [21]. Extracellular Cathepsin B is associated with amyloid plaques, and colocalizes with Aβ in regulated secretory vesicles in chromaffin cells in AD brains [22]. One report demonstrated that Cathepsin B reduces the relative abundance of Aβ through limited proteolysis, suggesting that the activation of Cathepsin B could offer a therapeutic strategy for AD [23]. Neprilysin is another major extracellular Aβ degrading enzyme that clears Aβ from the brain, and the injection of human neprilysin decreased amyloid plaques in the transgenic mouse [24]. AD therapeutic options also include acetylcholinesterase inhibitors and the *N*-methyl-d-aspartate receptor antagonist memantine, a glutamate receptor associated channel blocker, although there are side effects in the treatment of AD [25].

Glutathione is an antioxidant in brain cells. It reacts with ROS and oxidized products forming gluthathione disulphide. Vitamin E is another endogenous antioxidant that protects against lipid peroxidation, and high levels of vitamin E have been shown to be related to a reduced risk of AD [26]. Vitamin C is a water-soluble antioxidant that is necessary for the reactivation of vitamin E. Although vitamins C and E have been used in clinical applications to prevent AD, no clear beneficial effect has been demonstrated [2].

## 4. Stem Cell Treatment for AD

Stem cells include embryonic stem cells (ESCs), induced pluripotent stem cells (iPSCs), and tissue-derived stem cells, such as bone marrow (BM)- and adipose-derived stem cells. Stem cell-derived neurons have the potential to integrate into the existing neural networks of the host brain [27]. And stem cell transplantation appears to increase acetylcholine levels to improve cognition and memory in the animal model [28]. Moreover, stem cells secrete neurotrophic factors to modulate neuroplasticity and neurogenesis [29,30].

ESCs are self-renewing, totipotent cells that can differentiate into neuron progenitor cells (NPCs) *in vitro*, and this can result in a therapeutic effect when these cells are transplanted into AD animal models. ESC-derived NPCs were transplanted into an Aβ-injured rat model and the escape latency was significantly increased compared to phosphate buffered saline (PBS)-treated controls 2 weeks after the Aβ injection. The Morris water maze test was performed 16 weeks after transplantation, at which time the escape latency was found to have significantly decreased when compared to sham controls. Moreover, ESC-derived NPCs have been reported to be able to differentiate into astrocytic and neuron-like cells *in vivo*. These results suggested that ESC-derived NPCs ameliorated memory impairment. Although ESCs result in teratoma formation, it has been shown that ESC-derived NPCs can treat neurodegenerative diseases [31].

Human iPSCs were derived from skin cells by retroviral expression of octamer-binding transcription factor 4 (OCT4), sex determining region Y-box 2 (SOX2), proto-oncogene proteins c myc (cMYC), and kruppel-like factor 4 (KLF4), and can differentiate into neural cells. One report showed that induced iPSCs were generated from fibroblasts of familial ADs, and that these iPSCs differentiated into neurons that may increase Aβ42 secretion. Moreover, Aβ secretion from these differentiated neurons was affected by γ-secretase inhibitors, suggesting that these neurons have a pharmacological response to γ-secretase inhibitors. Thus these iPSCs may provide a potential strategy for the development of drugs against AD [32].

Mesenchymal stem cells (MSCs) are multi-potent progenitor cells that are mainly isolated from BM [33], adipose tissue [34], and the umbilical cord [35]. MSCs have been shown to differentiate into osteoblasts, adipocytes, and pancreatic islets [36,37]. BMMSCs have been reported to have the ability to modify and influence almost all the cells of the innate and adaptive immune systems mediated by BMMSC-soluble factors, including IL-6, IL-10, TGF-β, and PGE_2_ [38,39,40]. BMMSCs strongly inhibited the maturation and functioning of monocyte-derived DCs by interfering selectively with the generation of immature cells via the inhibitory mediator of MSC-derived PGE_2_. BMMSCs have also been shown to alter the natural killer (NK) cell phenotype, and suppress the proliferation and cytokine secretion of NK cells [41,42].

BMMSCs were able to home in on the injured brain and increase the number of positive cells for choline acetyltransferase. Furthermore, BMMSCs were able to remove Aβ plaques from the hippocampus and to reduce Aβ deposits through the activation of endogenous microglia in an induced AD mouse model [43,44]. Moreover, human MSCs enhance autophagy, promote Aβ clearance and increase neuronal survival in an Aβ-treated mouse model [45]. The transplantation of human MSCs has reduced infarct size and improved functional outcome, and autologous BMMSCs have been transfused to brain ischemic disease patients in clinical application [46]. In contrast, adipose-derived stem cells (ADSCs) were isolated from the inguinal fat pads of rats, and induced to differentiate into neurons or astrocyte-like cells *in vitro*. The transplantation of ADSCs allowed them to differentiate into neuron-like and astrocyte-like cells around the hematoma, accompanied with up-regulation of vascular endothelial growth factor expression and improvement of neural function, suggesting that ADSCs benefit neural differentiation and induce functional improvement in the rat [47]. When human ADSCs were intravenously injected into an AD mouse models, these cells could be found in the brain up to 12 days after their injection [48]. One report suggested that, when transplanted into the brain, adipose-derived MSCs (AMSCs) improved Ach levels as well as cognitive and locomotor functions in aged mice, and modulated microglia activation [29,49]. Human cord blood-derived stem cells have been shown to improve neuropathological and behavioral recovery from acute spinal cord trauma [50]. Human umbilical cord-derived MSCs can be induced to differentiate into neuron-like cells, and these cells were transplanted into an amyloid-β precursor protein (AβPP) and PS1 (AβPP/PS1) transgenic AD mouse model. The cognitive function was improved and Aβ deposition was reduced after transplantation. These beneficial effects were associated with the activation of M2-like microglia [51,52].

## 5. Intra-Bone Marrow-Bone Marrow Transplantation (IBM-BMT) and AD Mouse Model

The MRL-MpJ-lpr/lpr (MRL/lpr) mouse is a mouse model for autoimmune diseases, and this strain shows abnormal radioresistant stem cells [53]. We have reported that BMT plus bone grafts can prevent the recurrence of autoimmune diseases, though this method failed in MRL/lpr mice because these mice are more radiosensitive after the onset of lupus nephritis [54]. Moreover, we reported that BMMSCs can become trapped in the liver and lung when BM cells are injected intravenously. We thus injected whole BM cells directly into the bone marrow cavity, as in IBM-BMT, for the treatment of autoimmune diseases in MRL/lpr mice. IBM-BMT, which not only replaces hematopoietic stem cells but also MSCs, has been proven to be the best method for allogeneic BMT because hematopoietic recovery is rapid, since the stroma cells directly home to the bone cavity and the restoration of T cell functions is complete even in donor-recipient combinations across the MHC barriers [55]. Up to this point, we have used IBM-BMT to successfully treat autoimmune diseases, osteoporosis, and Alzheimer’s disease [56,57,58].

### 5.1. IBM-BMT and Senescence-Accelerated Mouse Prone 8 (SAMP8)

The senescence-accelerated mouse (SAM) was established via the selective inbreeding of the AKR/J strain of mice by Takeda *et al.* at Kyoto University. According to a graded score for senescence, life span, and pathologic phenotype, there are nine SAM-prone (SAMP) strains including SAMP1-3 and SAMP6-11, and three SAM-resistant strains (SAMR) including SAMR1, 4, and 5. These mice show age-related deficits in learning and memory and impaired immune response. Moreover, oxidative stress has been shown to be associated with mitochondrial dysfunction, and induced the excessive production of ROS and neurodegeneration in these two strains [59]. Heme oxygenase (HO) is an enzyme that catalyzes the degradation of heme. There are three isoforms: HO-1, HO-2 and HO-3. HO-1 is an inducible isoform, in responding to stress such as oxidative stress, hypoxia, and heavy metals. HO-2 is a constitutive isoform that is expressed under homeostatic conditions. HO-1 was reported to respond to excessive amyloid provocation and mitochondrial insufficiency, presenting chronic over-expression in the AD brain [60]. The SAMP8 was first reported to have age-related occurrence of deficits in learning and memory in 1986. SAMP8 mice showed significant impairment in the passive avoidance response, active avoidance tasks and spatial learning tasks when compared with age-matched SAMR1 [61]. Neuropathological studies showed that Aβ deposition was similar to that in AD in humans. Aβ levels in the hippocampi of SAMP8 increased with aging, as shown by the enzyme-linked immuosorbent assay method, although no senile plaque-like structures were found. However, Aβ-like deposition increased with aging, as was confirmed by immunoblot analysis. Some Purkinje cells seemed to disappear with aging in the medial cerebellum and vermis of SAMP8, which was consistent with the finding in AD brains. Thus the SAMP8 is shown to be an acceptable rodent model for AD [62,63,64,65]. Moreover, Aβ has been shown to play a central role in the pathophysiology of AD through the induction of oxidative stress. One report has demonstrated that antisense oligonucleotides directed against PS-1 in old SAMP8 mice improved learning and memory deficits and reduced Aβ-mediated oxidative stress [66]. Another report indicated that hydrocotyle sibthorpioides administration prevented declines in spatial learning and memory by the scavenging of free radicals, up-regulating the activity of antioxidant enzymes, decreasing the level of Aβ and ameliorating dysfunction in synaptic plasticity in SAMP8 mice [67].

**Figure 1:**
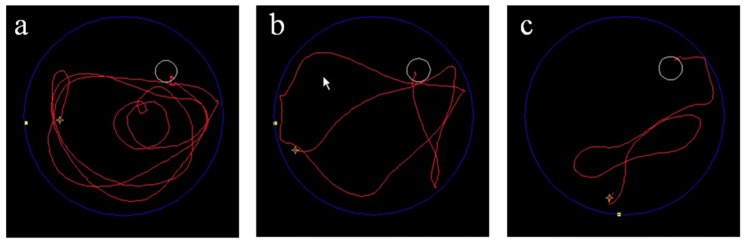
Escape latency of Senescence-Accelerated Mouse Prone 8 (SAMP8). (**a**) Untreated SAMP8; (**b**) SAMP8 treated with intra-bone marrow-bone marrow transplantation (IBM-BMT); and (**c**) SAM-resistant strains 1 (SAMR1).

BM cells have been shown to increase the number of activated microglia, and to reduce amyloid deposits via phagocytosis of Aβ and thereby prevent the progression of AD [68]. We used allogenic IBM-BMT to transfer normal BM stem cells in SAMP8. The 4-month-old SAMP8 mice received fractionated irradiation twice a day (4.5 Gy × 2, with a 4 h interval). One day after the irradiation, whole bone marrow cells from 8-week-old C57BL/6 mice were injected into the recipient mice. These mice were then tested by the Morris water maze test, which is thought to be a sensitive assay for brain abnormalities, especially in the hippocampus [69]. We used it for examining the effects of IBM-BMT on spatial learning and memory ability. Three months after IBM-BMT, there was no significant difference between the escape latency and the swim speed of 4- and 7-month-old SAMR1. However, the escape latency of SAMP8 treated with IBM-BMT was significantly shorter than that of age-matched SAMP8 (Figure 1), although there was no difference in swim speed. The swim paths of SAMP8 treated with IBM-BMT were more directly toward the hidden platform than those of SAMP8. Analyses of the water maze tests showed the impairment of spatial memory in SAMP8 to have been ameliorated.

Proinflammatory cytokines are involved in the formation of neuritic plaques in Alzheimer’s disease (AD) [70,71]. RT-PCR results showed that the expression of IL-6, IL-1β and inducible nitric oxide synthase (iNOS) decreased, while TGF-β increased in the SAMP8 treated with IBM-BMT, compared to age-matched SAMP8. Western blot analysis to examine HO-1 expression showed a significant decrease in the ratio of HO-1 to actin in the SAMP 8 treated with IBM-BMT, compared to age-matched SAMP8 [58]. Our results suggested that IBM-BMT improved inflammation and oxidative stress in AD model mice.

### 5.2. IBM-BMT and SAMP10

SAMP10 show age-related deficits in learning and memory as well as emotional disorders, brain atrophy, shrinkage and loss of cortical neurons, accumulation of neuronal DNA damage, reduced hippocampal cholinergic receptors, decreased neurotrophic factors, increased sphingomyelinase, and elevated oxidative-nitrative stress. SAMP10 also showed decreased catecholamine synthesis in the cerebral cortex, related with the decline in learning and memory abilities with aging, and SAMP 10 have thus also been extensively used in studies as AD model mice [72,73]. In contrast, the microglia showed a shorter combined projection length, and there were fewer segments and tips in the SAMP10 than in the SAMR1, which is consistent with neuronal degeneration in the SAMP10. Moreover, the levels of age-related pro-inflammatory cytokines such as IL-1β and IFN-γ showed greater increases in the SAMP10 than SAMR1 [74].

The immune system modulates CNS function and behavioral process. The thymus is a source of T cells, affecting the adaptive immunity. The immune system plays a central role in modulating learning, memory and neural plasticity. Under normal quiescent conditions, immune mechanisms are activated by environmental/psychological stimuli and positively regulate the remodeling of neural circuits, promoting memory consolidation, hippocampal long-term potentiation and neurogenesis. These beneficial effects of the immune system are mediated by complex interactions among brain cells with immune functions (particularly microglia and astrocytes), peripheral immune cells (particularly T cells and macrophages), neurons, and neural precursor cells [75]. We investigated whether neurodegenerative diseases were associated with thymus dysfunction in SAMP10. We first analyzed the lymphocyte populations in the peripheral blood by fluorescence-activated cell sorting (FACS). Our results showed that the percentages of CD4 and B220-positive cells were significantly lower, but the percentages of CD11b/Gr-1 double-positive cells were significantly higher in the 24-week-old SAMP10 than in the 6-week-old SAMP10, although there was no significant difference in the percentage of CD8 positive cells between 6-week- and 24-week-old SAMP10. Moreover, the percentage of CD4/TNFα T cells in the spleen of 24-week-old SAMP10 was significantly reduced compared to that of 6-week-old SAMP10, suggesting that the immune system was damaged in the SAMP10 [76].

Normal aging is associated with anatomical and functional changes in both the thymus and bone marrow. The aged thymus shows a disproportionate loss of thymic epithelial cells (TECs) and disrupted thymic architecture [77,78], resulting in an increased risk of autoimmunity through the escape of potentially self-reactive T cells from the disrupted thymic microenvironment. Sirt1 is a class III histone deacetylase within the sirtuin family of related proteins that is uniquely dependent on nicotinamide adenine dinucleotide^+^ (NAD^+^) for catalysis. Sirt1 has been implicated in processes as varied as metabolism, differentiation, cancer, stress response and aging [79]. Sirt1 negatively regulates T cell activation and plays a major role in clonal T cell anergy in mice. Loss of Sirt1 function results in abnormally increased T cell activation and a breakdown of CD4^+^ T cell tolerance [80].

Our results showed that the thymus was significantly lighter and the percentage of CD4^+^CD8^+^ was lower in the 24-week-old SAMP10 than the 6-week-old SAMP10. The expression of keratinocyte growth factor (KGF), Aire and Sirt1 was decreased on the TECs of 24-week-old SAMP10. Our previous report suggested that bone marrow cells contain the precursors of functional TECs, and that they can differentiate into TECs, thereby correcting thymic function [81]. RT-PCR showed that downregulated KGF, Aire and Sirt1 on the TEC of 24-week-old SAMP10 were improved after IBM-BMT. Thus, the findings showed that the dysfunction on the TEC of 24-week-old SAMP10 was modulated by allogeneic bone marrow cells in this experiment, suggesting that BMT might improve TEC function in SAMP10 [76]. We will focus future studies on the affects of stem cell transplantation on the pathologic changes in brain. Another report examined the finding that IBM-BMT facilitates the entry of transplanted BM cells into the brain parenchyma. IBM-BMT may thus prove beneficial in the experimental treatment of psychiatric and neurological diseases [82].

## 6. Conclusions

**Figure 2:**
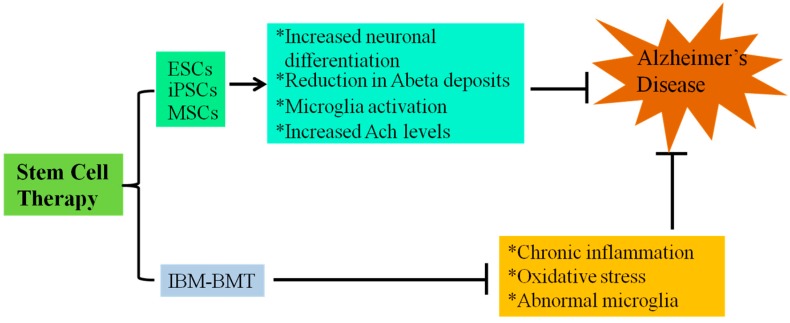
Summary of Stem Cell Therapy for AD.

Aging is the greatest risk factor for the onset of AD. For the most part, pharmacological interventions are aimed at relieving the symptoms of AD, but stem cell therapy not only has the potential to generate new neurons and replace damaged neurons but also to modulate the immune system (Figure 2).

With further clarification of the mechanisms by which AD progresses, stem cell therapies may well prove to be both safe and effective treatments. In time, more advanced stem cell therapies hold the potential for the clinical treatment of this debilitating disease.
